# Association of Variants in *COMT, RASSF1* and *GPM6A* with the Risk of Paranoid Schizophrenia Patients in Prof HB Saanin Psychiatric Hospital, West Sumatra, Indonesia

**DOI:** 10.21315/mjms2022.29.2.4

**Published:** 2022-04-21

**Authors:** Luluk YUNAINI, Edwina KHAIRAT

**Affiliations:** 1 Department of Medical Biology, Faculty of Medicine, Universitas Indonesia, Jakarta, Indonesia; 2 Master’s Programme in Biomedical Science, Faculty of Medicine, Universitas Indonesia, Jakarta, Indonesia

**Keywords:** paranoid schizophrenia, polymorphism, GPM6A, RASSF1, COMT

## Abstract

**Background:**

Schizophrenia is a multifactorial disease in which genetic factors play a greater role than other factors. The genes of importance in schizophrenia patients are the genes that encode for neurotransmitters associated with low minor allele frequency (MAF) scores. This study was aimed to determine the association of genetic variations in *catechol-O-methyl transferase (COMT)*, *Ras association domain family member 1 (RASSF1)* and *glycoprotein M6A (GPM6A)* with the risk of paranoid schizophrenia (PS) in patients admitted to Prof HB Saanin Psychiatric Hospital, West Sumatra, Indonesia.

**Methods:**

Genotyping analysis through polymerase chain reaction-restriction fragment length polymorphism (PCR-RFLP) and PCR-amplification refractory mutation system (ARMS) was performed in 100 PS patients and 100 healthy controls. Chi-square and Fisher’s exact tests were used to compare the frequencies of genotype and allotype between the PS and control groups. Odds ratio (OR) with 95% confidence interval (95% CI) were calculated to determine the relative risk of PS with respect to genetic variations.

**Results:**

Polymorphism rs13142920 in *GPM6A* was associated with significantly elevated risk of PS (*P =* 0.020; OR = 1.60 [95% CI: 1.08, 2.39]). However, *COMT* rs4680 and *RASSF1* rs2073499 polymorphisms were not significantly associated with PS.

**Conclusion:**

The *GPM6A* rs13142920 polymorphism holds great potential as a genetic marker in PS patients.

## Introduction

Schizophrenia is a psychiatric disorder with complex pathophysiology related to the development neurons (1). Early symptoms of this disorder usually appear in late adolescence or early adulthood. These disorders are distinguished by common psychotic symptoms, such as delusions and hallucinations, loss of interest and encouragement, changes in emotional reactivity and disorganised behaviour (2). In 2016, the World Health Organization (WHO) reported that more than 21 million people worldwide experience schizophrenia. According to Ministry of Health, Indonesia data from 2018, the prevalence of severe mental disorders, such as schizophrenia, in Indonesia was estimated to be around 1.6 million people or 7 per 1,000 inhabitants. This number has increased four-fold since the previous Ministry of Health consensus in 2013. West Sumatra ranked seventh out of 33 provinces with an increase in the number of schizophrenia cases between 2013 and 2018 (3). Schizophrenia affects 0.5%–1% of individuals across distinct ethnic populations (4).

Schizophrenia is classified into five types based on the predominant symptoms: i) paranoid schizophrenia (PS); ii) hebephrenic schizophrenia (HS); iii) catatonic schizophrenia (CS); iv) residual schizophrenia (RS) and v) unclassified schizophrenia (US). PS is characterised by delusions, hallucinations, uncertain anxiety, contentiousness and arguing and violent behaviour. HS is also known as disorganised schizophrenia or chaos. CS is characterised by symptoms of decreased reactivity to the environment. RS is characterised by flat feeling, withdrawal from social interactions, eccentric behaviour, illogical and irrational thoughts. US is characterised by general clinical features, such as delusions, hallucinations, incoherence and chaotic behaviour (5). The prevalence of PS is higher than other types of schizophrenia. Therefore, we focused on PS cases in this study. Furthermore, focusing on only one type of schizophrenia reduces the genetic bias that can arise when studying multiple types of schizophrenia (6–7).

Schizophrenia is a multifactorial disease in which both genetic and environmental factors interact (8). A high heritability of schizophrenia (about 80%) shows that genetic factors are more influential in schizophrenia than environmental ones (9). The candidate-gene approach has been used in several molecular studies on schizophrenia, which focus on genes that encode for proteins associated with schizophrenia pathophysiology, such as neurotransmitter dopamine (*catechol-O-methyl transferase* [*COMT*]), *Ras association domain family member 1* (*RASSF1*) and *glycoprotein M6A* (*GPM6A*) genes (10).

Three candidate genes were focused upon in this study: *COMT*, *RASSF1* and *GPM6A*. The *COMT* gene has been widely studied in different populations. It encodes the enzyme *COMT*, which is the main enzyme involved in the metabolism of the neurotransmitter dopamine (13). The rs4680, which is located in exon 4, is a variant in the *COMT* that has been linked to an increased risk of schizophrenia. In this variation, the gene undergoes a transversion, which leads to conversion of the amino acid valine to methionine. This alteration results in decrease in the activity of enzymes associated with the activity of the neurotransmitter dopamine, which is linked to symptoms of schizophrenia (14–15). The minor allele frequency (MAF) of rs4680 polymorphism in the general population is less than 10%, with 4% in the Japanese population, 6.9% in the Han Chinese population and 8% in the sub-Saharan African population (16). A genetic variant with a low MAF, especially one with MAF < 10%, might be considered as a candidate gene for disease.

The *RASSF1* and *GPM6A* genes were selected for this study based on the findings of genome-wide association study (GWAS), which revealed rs2073499 (A/G) and rs13142920 (A/C) polymorphisms in these genes, respectively, which were found to be associated with schizophrenia and had a MAF < 10% in East Asian population (12, 17–18).

The *RASSF1* rs2073499 polymorphism had an MAF 9.3% in the Han Chinese population and 9.7% in the Chinese population (17–18). The *RASSF1* gene encodes a protein that is associated with the RAS protein that modulates several growth-inhibiting responses and also acts as a tumour suppressor gene (19). It is hypothesised that increased expression of these tumour suppressor genes will result in excessive nerve cell death, which might impair the nervous system function associated with schizophrenia (20–21). rs13142920 is a polymorphism in *GPM6A* with an MAF of 5.8% in the Japanese population, 6.6% in the Chinese population and 7.1% in the Han Chinese population (18). This gene encodes the glycoprotein M6A protein, which is a transmembrane protein and a member of the proteolipid protein family that is most commonly expressed during nerve cell differentiation and development (22).

In Indonesia, several studies have been conducted to elucidate the relationship between genetic variation and schizophrenia. According to Rudianto et al. (23), the 8NRG433E1006 variant in the *NRG1* (Neuregulin-1) was not associated with Javanese schizophrenic patients. Sutrisna and Yulianti (24) found that the rs3213207 polymorphism is not significantly associated with schizophrenia among Javanese patients. So far, no candidate genetic markers for schizophrenia have been discovered in Indonesia. Hence, this study was aimed to determine the association of genetic variations in *COMT, RASSF1* and *GPM6A* with the risk of PS in patients admitted to Prof HB Saanin Psychiatric Hospital, West Sumatra, Indonesia.

## Methods

### Patient Characteristics and Ethical Approval

In this study, we used a case-control design with a total of 200 people, including 100 patients with PS at the Prof HB Saanin Psychiatric Hospital and 100 people as the controls who had no history of schizophrenia or any other mental disorders for at least three generations. The affected group included 83 males and 17 females aged 19 years old–64 years old, while the healthy controls were selected from the general public using a questionnaire and consisted of 26 males and 72 females aged 18 years old–39 years old.

### Blood Sample Collection

Two to three millilitres of whole blood was collected from every participant and stored in a storage tube containing the anticoagulant ethylenediaminetetraacetic acid (EDTA).

### DNA Extraction

DNA was isolated from whole blood using the salting out method that has been validated by Molecular Biology Laboratory of Biology Department, Faculty of Medicine, Universitas Indonesia. Briefly, 3 mL blood is added to a 15-mL tube and filled with red blood lysis solution in a ratio of 1:3. The mixture was incubated at room temperature for 10 min. Then, it was centrifuged at 1,500 rpm for 10 min and the supernatant was discarded. This step was repeated until the pellet was white. Next, 2 mL cell lysis solution (1 M Tris hydrocloric acid, 0.5 M EDTA and 10% sodium dodecyl sulfate (SDS) was added to the pellet and mixed until homogeneous. The mixture was incubated at 37 °C for 30 min. Then, 1.3 mL protein precipitation solution (5 M ammonium acetate) was added to the mixture and vortexed for 15 s–20 s. The mixture was centrifuged at 3000 rpm for 15 min. The supernatant was transferred into a tube that already contained 2.3 mL cold isopropanol. The tube was then inverted until DNA chromatin was visible. Then, the mixture was incubated at 37 °C for 2 h on water bath, followed by centrifugation at 3,000 rpm for 5 min. Next, 1.3 mL alcohol (70%) was added and then the tube was inverted. The supernatant was discarded and the tube was dried in an inverted position for 1 h. Finally, 300 μL Tris-EDTA (TE) buffer was added and the tube was incubated at 37 °C for 2 h on a water bath. The solution was then transferred to a 1.5-mL tube and refrigerated at −20 °C.

### DNA Amplification

The PCR technique was used to amplify the DNA target using the Gotaq^™^ PCR Core System Kit (Promega) according to the manufacturer’s protocol. The RFLP-PCR primer for the *RASSF1* was designed using the PrimerQuest online software, whereas the PCR-amplification refractory mutation system (ARMS) primers for the *COMT* and *GPM6A* were designed using the Primer1 online software. In order to prevent hairpins and dimers, the following primer criteria was selected: number of bases, 18 bp −30 bp; melting temperature (Tm), 52 °C– 58 °C; and guanine-cytosine (GC) percentage, 45%–60% (25). The National Centre for Biotechnology Information (NCBI)’s basic local alignment tool (BLAST) was used to confirm primer specificity. Table 1 contains detailed information about the PCR primers used in this study.

DNA samples were amplified for up to 30–35 cycles, beginning with a 5-min pre-denaturation temperature of 94 °C, followed by a cycle of denaturation at 95 °C for 30 s, annealing at 56 °C–69 °C for 30 s (Table 1) and elongation at 72 °C for 30 s. The extension phase comprised 72 °C for 7 min at the end of the cycle. The PCR product was verified using electrophoresis using 1.5%–2% agarose gel. Ethidium bromide was added to agarose gel for DNA visualisation with ultraviolet (UV) on UV longlife^™^ Filter Spectroline and photographed. The size of PCR product for each gene is listed on Table 1.

### Genotyping

#### Restriction Fragment Length Polymorphism

**Figure 1:**
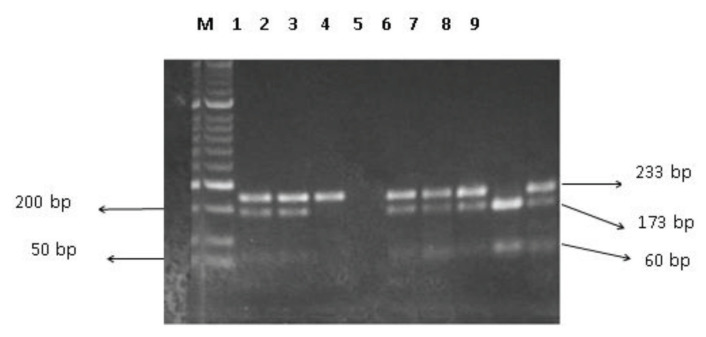
Agarose gel electrophoresis of PCR-RFLP products for the *RASSF1* rs2073499. Lane M represents 50 bp DNA ladder marker; lanes 1, 2, 5, 6, 7 and 9 represent GA genotype; lane 3 represents AA genotype; lane 8 represents GG genotype; and lane 4 represents negative control

*DrdI* enzyme (New England BioLabs) was used to perform RFLP for rs2073499 (G/A). The restriction reaction was performed in a 20-μL system, containing 1 unit of *DrdI*, 1 μg DNA fragment, 2 μL restriction enzyme (10×) buffer solution and remaining ddH_2_O. The DNA fragment was detected for their quality using 2% agarose. The restriction fragments produced by restriction enzymes were 173 bp and 60 bp long (Figure 1).

#### Amplification Refractory Mutation System

**Figure 2:**
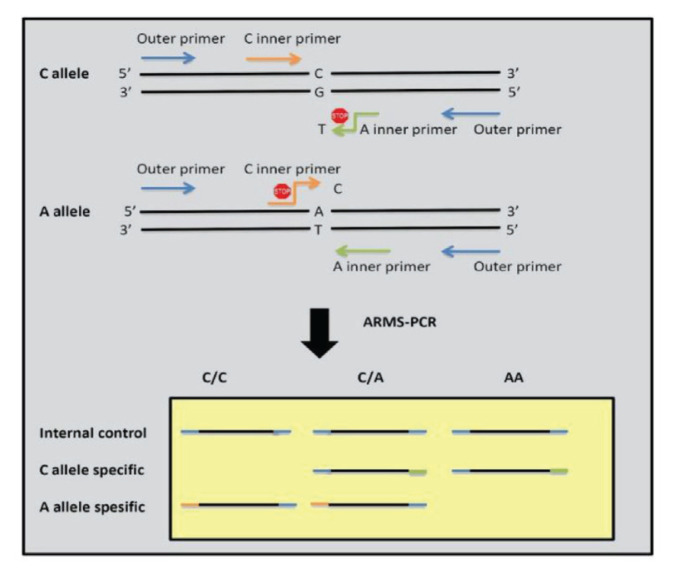
Tetra-primer PCR-ARMS method (26) (with modification). Different colours represent different primers involved in the PCR reaction. Blue: outer primer, orange: inner specific primer for C allele, and green: inner specific primer for A allele

**Figure 3:**
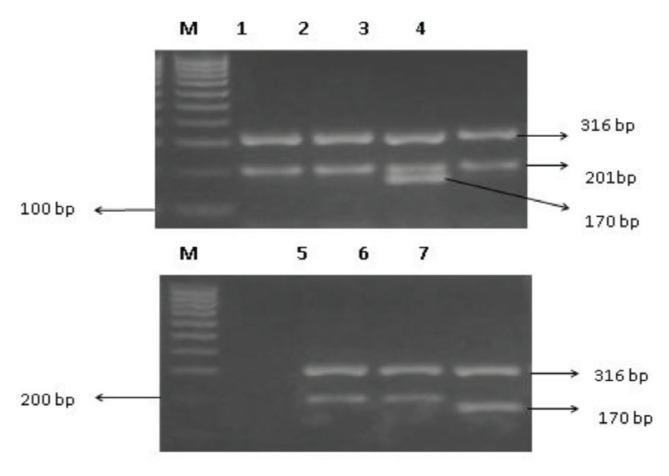
Agarose gel electrophoresis of ARMS-PCR products for the *GPM6A* rs13142920. Lane M represents 100 bp DNA ladder marker; lanes 1, 2 and 4–6 represent CC genotype; lane 3 represents CA genotype; and lane 7 represents AA genotype

**Figure 4:**
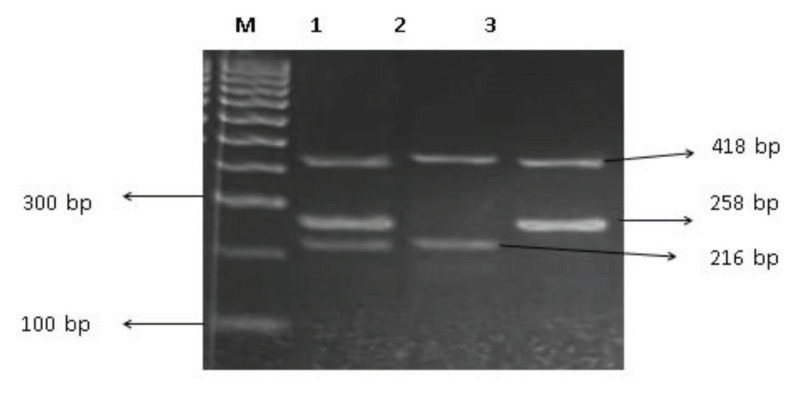
Agarose gel electrophoresis of ARMS-PCR products for the *COMT* rs4680. Lane M represents 100 bp DNA ladder marker; lane 1 represents GA genotype; lane 2 represents AA genotype; and lane 3 represents GG genotype

In ARMS, four primers were used to detect the rs4680 (G/A) and the rs13142920 (C/A) polymorphisms (Table 1). Each gene has one pair of internal control primers known as forward outer and reverse outer primers. For the detection of genetic variations in the *COMT* gene, reverse inner and forward outer primers were used for the G allele and forward inner and reverse outer primers were used for the A allele. Figure 2 depicts the PCR-ARMS method scheme (26). The PCR product of the *GPM6A* gene contained three DNA fragments with sizes of 316 bp for internal control, 201 bp for C allele-specific primers and 170 bp for A allele-specific primers (Figure 3). The PCR product of the *COMT* gene contained three DNA fragments with sizes of 418 bp for internal control, 258 bp for G allele-specific primers and 216 bp for A allele-specific primers (Figure 4).

### Statistical Analysis

Statistical analysis was performed to determine the association between genotype and allotype frequency in PS group using the Chi-square or the Fisher’s exact tests, while the odds ratio (OR) was used to determine the significance of genetic variations in schizophrenia disease manifestations. Statistical Package for the Social Science (SPSS) software version 25 was used for statistical analysis.

## Results

Table 2 shows the genotype distribution of the *COMT*, *RASSF1* and *GPM6A* genes. Statistical analysis revealed that the *GPM6A* polymorphism was significantly related to the risk of PS (*P* < 0.05; OR (CA and AA) = 2.92; CI 95%: 1.47, 5.81). To determine which genotypes were risk factors for PS, additional analysis was performed using dominant genetic modeling (Table 3). Individuals with the CA and AA genotypes had a *P*-value < 0.05, an OR > 1, and a CI > 1, indicating that they were at a higher risk of schizophrenia than those with the CC genotype. However, there was no significant association between rs4680 and rs2073499 genotypes and PS.

The Hardy-Weinberg analyses for the *RASSF1*, *GPM6A* and *COMT* genes in both the case and control groups are shown in Table 2. In two groups, the *RASSF1* and *COMT* genes exhibited non-significant *P*-values for Hardy-Weinberg equilibrium. This result indicated that the observed versus expected genotype distribution are comparable, or that the population is in Hardy-Weinberg equilibrium. Surprisingly, the affected group exhibited a significant *P*-value for the *GPM6A* genotype distribution, which was not in Hardy-Weinberg equilibrium.

Table 4 also shows the allotype distribution of the *COMT*, *RASSF1* and *GPM6A* genes. The statistical test revealed that the allotype of the *GPM6A* gene exhibited a significant association with PS (*P* < 0.05; OR (A allele) = 1.60; 95% CI: 1.08, 2.39). In the West Sumatran population, the A allele was found to be more likely to be associated with schizophrenia than the C allele. *COMT* and *RASSF1* allotypes, on the other hand, exhibited no significant association with PS.

## Discussion

Our findings revealed that there was no significant association between the rs4680 and rs2073499 genotypes and allotypes and the risk of PS. The results pertaining to *COMT* gene variant were similar to those of the studies done on Asian populations reported in Korea (27), Taiwan (28) and France (13). However, in other ethnic Asian populations, such as Saudi Arabia (31), India (32), China (30) and Turkey (29), a significant association has been reported between genotype and allotype frequencies in schizophrenia group compared to the control group.

Previous studies have also shown that the rs4680 polymorphism is associated with schizophrenia risk (33–34), which warrants the need to assess the other populations belonging to different ethnicities. Furthermore, the rs4680 variant in exon 4 is linked to the amino acid change from valine to methionine, with the val/val genotype associated with higher enzyme activity and less dopamine than the met/met genotype. As a result, the val/met heterozygous genotype exhibits more stable enzyme activity and dopamine levels (35). Changes in dopamine levels in the prefrontal cortex are associated with both positive and negative symptoms of schizophrenia (36–37), with dopamine hyperactivity associated with positive PS symptoms and vice versa (30, 38).

Previous studies on the association between rs4680 and schizophrenia in some populations have yielded conflicting results. This variation in the results could be attributed to schizophrenia being a polygene disease (35). Our findings showed that rs4680 is not a candidate genetic marker of schizophrenia in the population from West Sumatra, Indonesia.

Our results also showed that rs2073499, similar to rs4680, was not significantly associated with PS. This finding differs from previous GWAS studies in East Asia, including Indonesia (12) and China (39), and is associated with ethnic divergence. In this study, we focused on a more specific population in West Sumatra, with Minang being the dominant ethnic group.

The *RASSF1* gene encodes a protein that belongs to the RAS family and plays a role in the apoptotic pathway. This protein functions as a tumor suppressor, suppressing proliferation and increasing apoptosis. According to Catts and Catts (20), an increased risk of schizophrenia is associated with increased expression of tumor suppressor genes, such as p53. It is assumed that variation in the *RASSF1* gene increases apoptosis of excess nerve cells, resulting in decreased nerve cell function and association with schizophrenia.

In this study, we observed that rs13142920 was significantly associated with PS (*P*-value = 0.007; 95% CI: 0.17, 0.68). When compared to the CA and AA genotypes, the OR of the CC genotype was 0.34, indicating that it had a low probability of being associated with schizophrenia manifestations (Table 2). This result was related to the OR of the C allele, which was lower than that of A allele. These finding indicated that the A allele had dominant effect on schizophrenia manifestations. The Hardy-Weinberg equilibrium analysis revealed that the rs13142920 genotype for the patient group has a significant value. Our findings suggested that the *GPM6A* variant might contribute to schizophrenia risk.

These findings are also supported by GWAS studies conducted by Lam et al. (12) and Ma et al. (40), which demonstrated that the *GPM6A* could be a candidate gene worth further research for mental illnesses, such as schizophrenia. Ma et al. (40) discovered a decrease in *GPM6A* expression in the hippocampal area in the schizophrenic patients. An association study on the rs10520303 polymorphism has also been conducted by Boks et al. (41). They reported that there was a significant association between this polymorphism and schizophrenia with depressive sub-phenotypes. This finding could be attributed to stressor regulatory activity of the *GPM6A*, which might result in a change of expression, thus affecting the function of M6A proteins in nerve cell differentiation and development. As a result, changes in the function of nerve cells in the hippocampus are associated with the emergence of schizophrenia symptoms (42–43). As previously report, the rs13142920 polymorphism is located in an intronic area of *GPM6A* that has an associated to schizophrenia and is likely to be linked to causative variants that can directly affect the appearance of schizophrenia by acting as an enhancer in the transcription process (44). Furthermore, variation in the intronic region directly adjacent to the exon can affect the accuracy of the splicing process (45).

## Conclusion

In conclusion, the rs13142920 polymorphism of *GPM6A* is a candidate genetic marker for PS patients in West Sumatra, Indonesia. Meanwhile, the rs4680 polymorphism in *COMT* and rs2073499 polymorphism in *RASSF1* do not hold great potential candidates for genetic marker for PS in West Sumatra. As each ethnicity has different genotype and allotype frequencies, it is suggested that the ethnicity of our cohort might influence our findings. Furthermore, schizophrenia is a polygenic disease with many genes influencing the pathogenesis of the disease as well as a number of genotype and allotype frequencies in each population. Therefore, further investigations on *COMT, RASSF1* and *GPM6A* on larger population are warranted to validate our findings and further elucidate the association between schizophrenia and variations in these genes.

## Figures and Tables

**Table 1:** PCR primer sequences corresponding to each candidate gene

Gene/Polymorphism	Primer sequences	PCR product (abp)	Annealing temperature (°C)	GC content (%)
*RASSF1/*rs2043799	F: 5′ GCTGGCTCCATACAGGAGTG 3′	233	60	60
	R: 5′ GGCTTGTGGTAGACCTGAGC 3′			60

*COMT/*rs4680	FI (A) : 5′ CCAGCGGATGGTGGATTTCGCTGTCA 3′	216	69	62
	RO: 5′ CTGAGCTGCTGGGGGGGTCTTTCCTCAG 3′	418		
	FO: 5′ TCTCTCCACCTGTGCTCACCTCTCCTCCG 3′			
	RI (G): 5′CGGGTCAGGCATGCACACCTTGTCCTTAAC3′	258		64

*GPM6A/*rs13142920	FI (C): 5′ TCTTTCGATTGCAAAGAATAGAGATTTAC 3′	201	56	38
	RO: 5′ AGCAATCTACGACTTGTAAGTCGTGAAT 3′	316		
	FO: 5′ AATATACAGTTGATTCAGCTTCGACTCAC 3′			
	RI(A): 5′ CTGCCCCATCTTTCAGCTACTCTAGT 3′	170		39

Note:

a Base pair

**Table 2:** The genotype frequencies of polymorphisms in *RASSF1*, *GPM6A* and *COMT* in PS and control groups

Polymorphisms	Ref/Alt	Genotype	Frequency		Hardy-Weinberg equilibrium		*P*-value	cOR (95% CI)

			PS (*n* = 100)	Control (*n* = 100)	Patients *P*-value	Control *P*-value		
*RASSF1* rs2073499	G/A	GG	0.13	0.17	0.458	0.752	0.358^a^	
		GA	0.54	0.44				
		AA	0.33	0.39				
*GPM6A* rs13142920	C/A	CC	0.15	0.34	0.000*	0.051	**0.007** a *	**2.92 (1.47, 5.81)**
		CA	0.73	0.58				
		AA	0.12	0.08				
*COMT* rs4680	G/A	GG	0.59	0.51	0.831	0.509	0.508^b^	
		GA	0.37	0.44				
		AA	0.04	0.05				

Notes: ref/alt = reference/alteration;

a Chi-square test;

b Fisher’s exact test;

c Odds ratio;

* indicates significant difference

**Table 3:** Combined genotype frequencies of the *RASFF1*, *GPM6A* and *COMT* in PS and control groups

No	Gene	Polymorphism	Genotype	Genotype Frequency				*P*-value	aOR (CI 95%)

				Patients		Control			

				Genotype I	Genotype II	Genotype I	Genotype II		
1	*RASSF1*	rs2073499	GG versus GA + AA	0.07	0.43	0.09	0.41	0.428^b^	**2.92 (1.47, 5.81)**
			AA versus GG + GA	0.17	0.33	0.20	0.30	0.377^b^	
2	*GPM6A*	rs13142920	CC versus CA + AA	0.07	0.42	0.17	0.33	**0.002** b *	
			AA versus CA + CC	0.06	0.45	0.04	0.46	0.346^b^	
3	*COMT*	rs4680	GG versus AA + GA	0.30	0.20	0.23	0.25	0.256^b^	
			AA versus GG + GA	0.02	0.48	0.03	0.49	1^b^	

Notes:

a Odds ratio;

b Chi-square test;

* indicates significant difference

**Table 4:** The allotype frequencies of polymorphisms in *RASSF1*, *GPM6A* and *COMT* in PS and control groups

Gene polymorphisms	Ref/Alt	Allotype	Frequency		*P*-value	aOR (CI 95%)

			PS (*n* = 200)	Control (*n* = 200)		
*RASSF1* rs2073499	G/A	G	0.40	0.39	0.838	
		A	0.60	0.61		
*GPM6A* rs13142920	C/A	C	0.52	0.63	0.020^b^*	0.62 (0.42, 0.93)
		A	0.49	0.37		1.60 (1.08, 2.39)
*COMT* rs4680	G/A	G	0.78	0.73	0.297	
		A	0.23	0.27		

Notes: ref/alt = reference/alteration;

a Odds ratio;

b Chi-square test;

* indicates significant difference
